# Phage Therapy—Challenges, Opportunities and Future Prospects

**DOI:** 10.3390/ph16121638

**Published:** 2023-11-22

**Authors:** Beata Zalewska-Piątek

**Affiliations:** Department of Molecular Biotechnology and Microbiology, Chemical Faculty, Gdańsk University of Technology, Narutowicza 11/12, 80-233 Gdańsk, Poland; beazalew@pg.edu.pl; Tel.: +48-58-347-1862; Fax: +48-58-347-1822

**Keywords:** phage therapy, regulatory framework, phage preparation, cultivation, purification, therapeutic phage products, advantages and disadvantages of phages

## Abstract

The increasing drug resistance of bacteria to commonly used antibiotics creates the need to search for and develop alternative forms of treatment. Phage therapy fits this trend perfectly. Phages that selectively infect and kill bacteria are often the only life-saving therapeutic option. Full legalization of this treatment method could help solve the problem of multidrug-resistant infectious diseases on a global scale. The aim of this review is to present the prospects for the development of phage therapy, the ethical and legal aspects of this form of treatment given the current situation of such therapy, and the benefits of using phage products in persons for whom available therapeutic options have been exhausted or do not exist at all. In addition, the challenges faced by this form of therapy in the fight against bacterial infections are also described. More clinical studies are needed to expand knowledge about phages, their dosage, and a standardized delivery system. These activities are necessary to ensure that phage-based therapy does not take the form of an experiment but is a standard medical treatment. Bacterial viruses will probably not become a miracle cure—a panacea for infections—but they have a chance to find an important place in medicine.

## 1. Introduction

Nowadays, the lowering effectiveness of antibiotics, as well as their overuse, favors the emergence of resistant pathogens. Environmental and nosocomial infections caused by multi-drug-resistant (MDR) bacteria are now a major threat to public health. Antimicrobial resistance (AMR) accounts for approximately 700,000 deaths per year [1,2]. However, it is assumed that this number will increase further in the coming years, given that the U.S. Food and Drug Administration (FDA) has approved only two new classes of antibiotics against Gram-positive bacteria (with little effect on Gram-negative bacteria) in the last 20 years [3]. Moreover, according to the Centers for Disease Control and Prevention (CDC) data, over 2.8 million MDR infections occur each year in the United States (U.S.), killing more than 35,000 people [4]. In turn, the World Health Organization (WHO) estimates that these infections will be the leading cause of 10 million deaths worldwide by 2050 [5].

Additionally, in 2022, the first systematic analysis was developed, which can provide an assessment of the global burden of AMR based on data from 2019 (i.e., literature reviews, hospital systems, surveillance systems, and others, covering 471 million individual records or isolates and 7585 study-location years). Considering 23 pathogens and 88 pathogen-drug combinations in 204 countries (mainly low- and middle-income), it was shown that 4.95 million people died due to bacterial AMR. Regardless of age, the highest mortality rate was found in Sub-Saharan Africa and the lowest in Australasia [6]. Respiratory infections caused 1.5 million resistance-related deaths resulted. The priority pathogens identified by WHO (including leading pathogens such as *Escherichia coli*, *Staphylococcus aureus*, *Klebsiella pneumoniae*, *Streptococcus pneumoniae*, *Acinetobacter baumannii*, and *Pseudomonas aeruginosa*) were responsible for 929,000,000 deaths attributable to AMR and 3.57 million deaths related to AMR in 2019 [6,7].

The increasing prevalence of MDR organisms requires the search for innovative non-antibiotic therapeutic methods and strategies. Current efforts are aimed at the widespread development and use of phages for treatment of human or animal infectious diseases as a new-old anti-bacterial therapeutic option [8,9,10,11,12,13]. The first reports on phages date back to 1896. Namely, Ernest Hankin, an English bacteriologist, demonstrated the presence of an unidentified substance with antibacterial activity against *Vibrio cholerae* in water samples from the Ganges and Jamuna Rivers [14,15]. In 1915, the British bacteriologist Frederick Twort independently hypothesized that ultramicroscopic viruses could act as antibacterial agents. For financial reasons, the discoverer did not continue his research. Therefore, the official discoverer of phages is considered to be Felix d’Herelle, a French-Canadian microbiologist from the Pasteur Institute in Paris, who coined the term viral bacteria eaters based on the two words bacterium and phagein [16,17,18].

In the post-antibiotic era, phage therapy (PT) (defined as the treatment of bacterial infections with bacteriophages), especially in a personalized form closely tailored to the patient’s needs, can be an alternative remedy used to treat human infections (resistant to available antibiotics and often fatal) [19]. This may be a link between the failure of antibiotics and clinical trials. Furthermore, given the emergence of zoonotic pathogens in the food chain, the use of phages to combat them is fully justified. Among the bacterial pathogens associated with poultry and swine infections, *E. coli* (colibacillosis), *Salmonella* spp. (gastrointestinal infections), *Campylobacter* spp. (campylobacteriosis), *Clostridium* spp. (necrotic enteritis), and *Listeria* spp. (listeriosis) predominate [13,20]. The studies carried out on livestock indicate their high efficacy, which is important for the protection of the health of both humans and animals [13]. This is also an important aspect in terms of meeting demand for meat as the world’s human population increases (e.g., 120.5 million tonnes of meat in 2018) [21].

The goal of PT as a viral delivery method is to kill the pathogenic bacterial strain(s) without disturbing the balance of the natural microflora of the treated patient. Phages show the ability to multiply in the target bacteria as long as their cells are present in the diseased organism. It most often involves the use of lytic and engineered phages, phage proteins, or phages and antibiotics. Once they reach the site of infection, there is an exponential increase in the number of phage particles. As a result, re-administration of phages is not required to achieve the desired therapeutic effect. The killing of the bacteria is accompanied by a decrease in phage titers until they are completely eliminated from the patient’s body with urine [11,22,23].

The pharmacokinetics (PK) and pharmacodynamics (PD) of PT pose a major problem for clinical trials. To define PK, a dose representing the number of phage particles administered is needed. Key factors influencing PK include phage absorption, biodistribution, metabolism, and elimination [23]. Adsorption is mainly dependent on the route of phage administration (e.g., intravenous, oral, or inhalation for blood, gastrointestinal, and respiratory infections, respectively) [24,25,26]. The size of the phage and its morphology (associated with phage uptake by cells) and the cell types at the site of administration can also have an impact on adsorption [27]. Similarly, the size of the phage, its morphology, different organs and cell types (e.g., blood, heart, lungs, kidneys, bladder, skeletal muscles, bone marrow, thymus, salivary glands, and brain), and, in addition, the microbiome strains (having the same or similar phage receptors) affect phage biodistribution within the body [24,28]. In turn, metabolism or metabolic inactivation of phages is influenced by environmental pH (the main problem with orally administered phages), the immune response (i.e., phagocytosis of phages mainly in the liver by Kupffer cells and stimulation in the spleen of the production of phage-neutralizing antibodies that reduce the antibacterial activity of the phage preparation), and the host microbiome (modulating phage load in the body) [29,30]. The elimination of the phage by the patient’s kidneys is strictly dependent on the size of the phage. Differences in phage excretion with urine were observed depending on the age of the patient (e.g., when administered orally, the phage detection rate was 35% and 87.3% in adults and children, respectively) [23,24,31].

PD, in turn, is related to the antibacterial activity of phage preparations. It is based on the analysis of multiplicity of infection (MOI), passive or active type of PT (PPT/APT), and innate and adaptive immune response (IR). MOI is defined as the ratio of the number of phages (infectious agents) added per bacterial cell (i.e., target), which does not always correspond to the number of phages interacting with pathogen cells. This is an important factor in preventing the development of phage resistance [32]. PPT and APT are related to the inundation threshold concept, which refers to the minimum phage concentration required to reduce the bacterial load. Therefore, treatment is passive when, without phage replication, the dose reaches the inundation threshold. This is influenced by bacterial activity as well as the development of bacterial resistance to phages. However, APT is dependent on phage replication to reach the inundation threshold. The efficacy of both of these therapies is influenced by the phage binding rate and latency period. In active therapy, the burst size is also important [33,34]. The administration of phages also induces innate (i.e., macrophages, neutrophils, and the complement system, enhancing antimicrobial activity) and adaptive responses (the production of phage-neutralizing antibodies depending on the route of phage administration and duration of therapy, the site of infection, and the functionality of the host immune system) [23,35,36]. Certainly, the consequences of antibody production by the treated organism and its impact on the effect of PT (positive or negative) require further research [37,38].

Over the past five years, PT has been revitalized again due to more clinical trials (e.g., in the USA, Australia, or Europe) evaluating its efficacy against MDR bacteria [22]. Nevertheless, this therapeutic approach requires the constant updating of available phage preparations and the use of new phages. Despite the effective clinical application of such therapy as early as 1919 (i.e., antidisentric d’Herelle’s phage treatment at the Hospital des Enfants-Malades in Paris), bacteriophages are still not approved for treatment in many countries in western Europe [39]. The main reason is the lack of an appropriate legal and regulatory framework in Western medicine. However, patients suffering from invasive bacterial diseases are treated based on expanded access to experimental therapy in emergency cases according to the Investigational New Drug (IND) program under the approval of the U.S. FDA or European Medicines Agency (EMA). In addition, the IND program enables pharmaceutical companies to obtain approval to conduct clinical trials in humans. Then, patients who have failed antibiotic therapy can receive phage-based treatment [26,40].

## 2. Regulatory Framework and Some Aspects of the Current Situation Regarding the Use of Phage Therapy

The ethical and regulatory laws regarding the compassionate use of PT vary from country to country [41,42,43]. PT currently includes single phages or combinations of phages (“phage cocktails”) with a broad spectrum of activity. It also allows quick matching of a phage or phages to a bacterial isolate (from an infected patient) for individualized and specific treatment. There are also only a few PT centers worldwide (including, e.g., Georgia, Belgium, Poland, the USA, and Australia) that continue phage-based treatment and provide important information about the efficacy of this form of therapy and clinical trials [44,45,46].

Experimental PT has been used for decades at the Eliava Institute of Bacteriophage, Microbiology, and Virology (EIBMV, 1923) of the Georgian Academy of Sciences in Tbilisi (off-the-shelf fixed-phage products) and the Ludwik Hirschweld Institute of Immunology and Experimental Therapy (HIIET, 1952) of the Polish Academy of Sciences in Poland (Wroclaw). The therapy is based on the Helsinki Declaration of the World Medical Association and aims to treat patients for whom proven medical interventions do not exist or have been ineffective [14,42,47,48,49]. After Poland joined the European Union (EU) in 2005, Polish PT became a model treatment for other countries around the world. Then, the establishment of the Phage Therapy Center (PTC) by Prof. A. Górski (as part of HIIET, operating in accordance with legislative, ethical, and administrative requirements in EU countries and in the U.S.) coincided with a large increase in morbidity and mortality caused by antibiotic-resistant superbugs worldwide [50].

In January 2018, the Belgian Minister of Public Health and the Federal Agency for Medicines and Health Products, FAMHP (as the Belgian competent authority for medicines), introduced legal regulations for the treatment of individual (specific) patients with personalized and non-standard preparations. The therapy is based on “magistral phage medicines”, also known as compounded prescription drugs in the U.S., routinely manufactured in the pharmacy according to the physician’s prescription and the technical and scientific standards of the pharmaceutical art (Article 3 of Directive 2001/83 and Article 6 quarter, § 3 of the Law of 25 March 1964) [40]. Magistral preparations, in addition to adapting treatment (by doctors) to the patient’s needs, are also a condition for the development of innovative drugs that are not produced by commercial producers and do not exist on the pharmaceutical market. Thus, Belgium (as the only Western country) opened the door to phage drugs and a personalized, sustainable approach to PT according to the “Prêt-à-porter or sur-mesure” paradigm [40,51]. Then, in June 2018, the first Center for Innovative Phage Applications and Therapeutics (IPATH) bringing innovative research and clinical practice to the field of medicine was established in North America (at the University of California, San Diego School of Medicine). The center focused on PT for life-threatening infections in humans as part of the FDA’s compassionate use program. In addition, this institution introduced phage treatment to clinical trials in order to assess its effectiveness and wide application. The first clinical trial included cystic fibrosis patients shedding *Pseudomonas aeruginosa* [52]. Australia is also an ideal country for conducting randomized, controlled, phage-based clinical trials. The Australian Therapeutic Goods Administration (TGA) has taken over many of the EMA’s policies. Both natural and genetically modified phages are considered approved applications for gene transfer. This will allow sponsors to conduct as many clinical trials as they want without further assessment by the TGA. In addition to the Australian Phage Therapeutics Center, there are also expert clinical research organizations and generous tax incentives [53].

The key element in matching a phage to a target bacterium is the phage bank, which contains a large collection of phages that are fully characterized and ready to be used in a clinical setting against a variety of bacterial strains. The largest phage resources today are the American Type Culture Collection (ATCC) and Public Health England (PHE) collections, also including the National Collection of Type Cultures (NCTC). The Israeli Phage Bank seems very interesting, too. It contains 300 phages targeting 16 pathogenic bacterial species [54].

Creative Biolabs (CB), founded in 2005, is also very helpful in the development of PT and the discovery of phages targeting bacteria to find effective and safe treatments and improve people’s health. This biopharmaceutical company provides comprehensive services for building its own tests and processes and analyzes supporting products in pre-clinical and clinical development. A wide range of natural phages from clinics and environmental sources are screened by CB. As an alternative to natural phages, synthetic phages targeting specific bacteria are also obtained using various phage engineering strategies. Experienced scientists use a number of parameters to evaluate and then select the optimal phage cocktails [55,56].

Given the increasing prevalence of multi-drug-resistant organisms (MDROs), there is a need to constantly enrich and expand phage collections and banks with new phages. The global phage market is expected to reach USD 1.4 billion by 2026. Moreover, it is desirable to develop new procedures for the production, concentration, and purification of high-quality and safe phage preparations, including not only therapeutic phages but also reporter and labeled phages. The implementation of harmonized phage cultivation and purification in research laboratories, working to obtain phage preparations for medicinal purposes with extended access, may result in the future development of PT in Western Europe [57,58].

## 3. Selected Examples of Phage Preparations—Methodology

Bacteriophages are bacterial viruses that are widespread in the environment (where bacteria occur) from which they can be isolated (e.g., water samples from rivers and lakes, hospital waste, and sewage treatment plants) and enriched and manufactured for medicinal purposes [58,59,60]. Exploiting the potential of phages in global therapy requires a thorough refinement of methods and measures, ultimately leading to a finished medicinal product. PT centers worldwide should aim to develop a universal phage isolation and purification scheme.

Phages can interact with the cell walls of bacteria, infect them, and multiply inside their cells. In addition, they disrupt the metabolism of bacteria and cause their lysis [39]. Considering replication cycles, phages can be classified into two groups: lytic (virulent) phages and lysogenic (temperate) phages. The lytic cycle includes early expression of phage genes, replication of its genome, late expression of virion structure and assembly genes, assembly of phage particles, and lysis of bacterial host cells. The lysogenic cycle is associated with the integration of the phage genome into the bacterial chromosome, the establishment of the prophage stage, and replication with the genome of the host cell. The prophage can be activated under certain environmental conditions, resulting in the start of the lytic cycle and the lysis of the bacterium [11,14,61].

PT is mainly based on strictly lytic phages with host ranges for clinically important bacterial species. The bacterial viruses are considered safe and are also able to penetrate the complex structure of the biofilm produced by bacteria. Furthermore, these bacterial parasites are characterized by high specificity, which means that often one species of phage replicates in only one species of bacteria [62,63,64]. Thousands of phages are stored in research laboratories around the world. Moreover, many of them are poorly equipped to obtain safe phage preparations. In turn, biotechnology companies do not have easy access to phages from scientists. This means that most life-threatening infections are left untreated purely for logistical reasons [22].

The phage preparations obtained (after isolation and enrichment) must be highly purified and clinically safe for therapeutic applications in a potential patient [65,66,67]. Therefore, the main problem in the preparation of therapeutic phages is their separation from the remains of bacterial cells, such as endotoxins (i.e., lipopolysaccharides, LPS consisting of a lipid component, Lipid A, a core oligosaccharide and a long heteropolysaccharide chain, the O-specific chain representing the surface antigen), exotoxins, peptidoglycan, nucleic acids, flagella, and other non-cellular compounds (e.g., culture medium). Failure to remove these harmful ingredients may result in many undesirable symptoms, which are very harmful to human health and life, including inflammation, septic shock, and even sepsis [65,68,69,70]. For phages against Gram-negative bacteria, the major pyrogen is LPS, whose toxicity depends on a strong innate immune response, including cytokines, hence the need to exclude endotoxins from phage preparations [71]. Removal of endotoxins from a phage medicinal preparation often requires product dilution, which lowers the phage content as an active ingredient [72,73,74]. The amount of endotoxins is determined by the endotoxic unit (EU). This corresponds to an activity of 100 μg of *E. coli* lipopolysaccharide. The highest endotoxin threshold allowed by European Pharmacopeia was set at 5 EU/kg/h for most intravenous applications [72,75]. For comparison, the endotoxin content of distilled water is about 20 EU/mL [76]. Greater tolerance of endotoxins occurs with oral administration [77,78].

Strategies for endotoxin removal from phage materials for human or animal use may vary depending on the composition of the product being purified. Currently, there are many reports on the elimination of endotoxins from biological fluids [65,68,74].

### 3.1. Cultivation, Purification, and Concentration of Phage Products

The preparation of crude bacterial suspensions (bacterial lysates) starts by infecting the target bacterial strain with the specific phage. Then, the multiplication of bacteriophage on bacterial cultures is carried out at appropriate conditions (e.g., 37 °C for 8–18 h). The next step includes the determination of phage titers expressed as plaque-forming units (PFU) using the double-layer agar technique (agar overlay tittering) or spot plaque tittering. Finally, the crude bacterial lysate is subjected to a selected purification procedure [57].

One of the methods was based on sequential ultrafiltration of the crude bacteriophage suspensions (bacterial lysates) cultured from *E. coli* and *Pseudomonas aeruginosa* through a polysulfone membrane (30 nm in diameter) under increased pressure (1 bar). Then, the concentrated phage suspension was subjected to a sepharose 4B chromatograph. Finally, the partially purified material was chromatographed on Matrex Cellulofine Sulfate (a spherical cellulose matrix), allowing ion exchange and affinity interactions with a wide variety of viruses and macromolecules [72]. Differences in the interaction of the viruses with the cellulosic matrix can also be observed, which are not dependent on the size of the viruses, the presence or absence of a lipid envelope, or the type of genetic material. Therefore, some of the viruses tested (i.e., feline herpesvirus, human measles, human herpes simplex virus type 1, human parainfluenza type 3, feline calicivirus) showed ionic interactions with a Cellufine Sulfate phase, but others did not interact at all (i.e., human adenovirus type 8, murine leukemia virus, human polio virus type 1, and human echovirus type 8) or bound reversibly (human *respiratory syncytial virus*) [79]. The applied procedure allowed us to obtain an acceptable level of endotoxins in phage suspensions in the range of 0.4–7 EU/mL. Bacterial lysates purified by this method contained 1.4–2 EU/10^8^ *E. coli* T4 and 0.25 EU/10^8^ *P. aeruginosa* F8 phage particles, respectively [72].

Another method was associated with the extractive removal of endotoxin from phage preparations with water-immiscible solvents (such as 1-octanol and 1-butanol) [73]. The purification procedure of crude bacterial lysates included filtration through membrane filters (0.22 μm), supplementation by adding MgCl_2_, and extraction with organic solvent to obtain the two-phase mixture. Then, the collected aqueous phase with phage particles was dialyzed against 25% aqueous ethanol and subsequently against aqueous 0.15 M NaCl to remove organic solvents and alcohol, respectively. The endotoxins were transferred into the organic phase. The last stage of this method was membrane filtration, which allowed for the removal of macromolecules derived from the medium and bacteria and the concentration of phages. Initial endotoxin levels in phage lysates were between 10^3^ and 10^5^ EU/mL, while after organic extraction in the aqueous phase, they were 5.3 EU/mL. The contamination of the T4 bacteriophage lysate of *E. coli* was, on average, 2.8 EU/10^9^ PFU. However, the relative endotoxin content in the final products was in the range of 0.9–11 EU/mL. The versatility of extracting bacteriophage lysate with organic solvents was analyzed using two other bacteriophages, the *E. coli*-specific HAP1 phage and the *P. aeruginosa*-specific F8 phage. The applied procedure reduced the titers of lysates to 47 and 33% for HAP1 and F8, respectively, compared to the original titers. In contrast, the endotoxin levels in the lysates dropped to 14 and 8 EU/mL from 6 × 10^4^ and 3800, respectively. The final products contained between 7 and 8.9 EU/10^9^ PFU [73]. The above technology was developed by another team using a vacuum-based method to remove organic solvents [80].

Additionally, one recently developed approach focused on the removal of endotoxins by performing multiple low-speed centrifugations, microfiltration, and cross-flow filtration (CFF) [57]. The presented production method allowed for high-titer phages (10^9^–10^12^ PFU), including liter-scale cultivation with a low endotoxin content (4.3–24.1 EU/mL). The above procedure was used for the purification of *Serratia* phage SM219; two *Klebsiella* phages, JG265 and JG266; and four *Pseudomonas* phages, PAK_P1, PAK_P5, E217, and PYO2. Phage lysates were subjected to sterilization by pressure-driven double dead-end filtration (inline 0.8-, 0.45-, 0.45-, and 0.22-μm membrane filtration) to exclude whole bacterial cells and cellular debris, and CFF (a pressure-driven scalable membrane filtration) with a molecular weight cutoff of 100 kDa to remove growth medium, endotoxins (~10 kDa in size), all known exotoxins (<30 kDa), peptidoglycans, flagella, and nucleic acids, and concentrate phage particles in phosphate buffer. Optionally, CsCl density gradient ultracentrifugation and dialysis were used to further confirm phage stock homogeneity. Ultimately, the residual endotoxins were removed by LPS-affinity chromatography. This procedure resulted in a 10^6^-fold reduction of endotoxins in purified phage preparations, with the concomitant obtaining of up to 64,000 therapeutic doses (at 10^9^ PFUs, the commonly prescribed intravenous dose) applicable in most cases of expanded-access phage therapy and phase I and II clinical trials [57,81]. Compared to other methods, the procedure described above does not require the use of chloroform and denaturing solvents to disrupt the bacterial cell wall and release internal phages, organic compounds and detergents for reduction of endotoxins in phage lysates, phage concentration and replacement of the growth medium (here by the use of CFF), and also specialized production equipment (such as a bioreactor) or a chromatography purification system [74,80,82,83]. The use of chloroform can cause denaturation of some phages as well as an increase in the amount of bacterial cell debris as a result of the lysis of phage-resistant bacteria (developing during long-term culture). The impact of this compound on human health (hepatitis, jaundice, and effects on the central nervous system after inhalation exposure) and animals (kidney and liver tumors) is also important [84]. In addition, the amount of chloroform in pharmaceutical products is strictly regulated, and according to the ICH (I/O Controller Hub) Q3C (R6) scientific guideline for residual solvent, the permissible daily exposure is 0.6 mg/day (concentration limit 60 ppm) and cannot be higher [85]. Summarizing, organic solvents and detergents are an effective method for reducing endotoxins in phage lysates. However, this is also a procedure that blocks the quantitative analysis of phage preparations based on chromogen and reduces the stability of phages during storage. A toxic effect of chloroform is also not excluded [73,74,80].

### 3.2. Genome Analysis and Characterization of Other Phage Properties

For expanded access to phage-based therapy, genome sequencing, analysis, and screening for lysogenic and harmful genes such as toxins, antibiotic resistance genes, and virulence factors are very important [86,87,88,89,90,91]. Sequencing allows us to quickly determine whether a phage is temperate or virulent. Viruses that cause lysogenization (i.e., temperate phages) of the bacterial host are generally avoided in PT. However, phages can be genetically modified to render them lysogenizable [90,92]. As part of bioinformatics analysis, it is also possible to identify genetic similarities with previously characterized phages, especially phages, for which previous therapeutic experiences have shown their beneficial or unfavorable effects [60,66]. It is necessary to ensure that the resulting phage preparation is safe for therapeutic use in humans and animals.

When characterizing phages used as therapeutic products, it is also important to determine the adsorption rate, latency, burst size, and thermal or pH stability (mainly as a basis for adjusting the phage production range and applications). Higher temperatures may extend the latent period of the phage, and lower temperatures (compared to optimal conditions) will limit the amount of phage genetic material introduced into bacterial cells [29,93]. The optimum pH for most phages is in the range 5–9 (with its maximum at about 5–6), while lowering the temperature allows the ph tolerance range to increase to 4–9 [29]. In order to classify and further analyze phages, their morphology must be examined. Observation by transmission electron microscopy (TEM) or other similar techniques provides a general classification position and relevant possible information about the phage of interest [59,94,95] (Table 1). In conclusion, it is important to consider not only what has been done in one’s own research, but also what other research teams have similarly achieved in terms of characterizing the phage in question.

## 4. Positive and Potentially Negative Features of Phage Treatment

Phage-based therapy can provide an alternative antibacterial strategy for the treatment of drug-resistant bacterial infections to commonly used antibiotics [19,58]. This type of treatment can also be individually adapted to each patient (infected with bacteria resistant to antimicrobial agents) as a personalized therapy. The procedure involves the isolation and analysis of a clinical pathogenic strain responsible for the disease process in a given patient, and then selecting a specific phage from the phage bank or extracted from the environment [81,96,97,98]. This is a very promising form of therapy, especially when the available antibiotic-based treatment strategies are exhausted. This is why it is so important to examine in depth the good and bad aspects of such therapy. All these activities will help to improve the treatment and safety profile of PT.

PT shows no visible side effects and is not pathogenic or harmful, as confirmed by studies in animal models and human clinical trials. The therapy has a similar outcome for both antibiotic-resistant and antibiotic-sensitive pathogens. Therefore, as a safe and effective procedure, it can be used to treat humans and animals, including MDR and chronic bacterial infections (such as biofilm-related and severe infections, e.g., sepsis, meningitis, pneumonia, osteomyelitis, septic arthritis, pyelonephritis, chronic nonhealing wounds, or periodontitis) [81,92,99,100,101,102,103,104,105,106,107,108,109,110,111].

### 4.1. Advantages

Naturally occurring phages provide many advantages over traditional antibiotics and the treatment based on them. These include low inherent toxicity in vivo, suitability for, e.g., patients with antibiotic allergies, and the inability to multiply in eukaryotic cells, which makes them safer than antibiotics [19,112,113]. In addition, phages are active independently of antibiotic resistance, and some phage-antibiotic combinations have synergistic antimicrobial effects (as confirmed by studies and clinical trials) [114,115]. The phages also show auto-dosing during infection (i.e., replication in vivo), in which the number of phages increases or decreases relative to the number of bacterial hosts [112,116,117]. This means that when there are no bacteria, there are also no viable phages. Bacterial viruses also replicate and can therefore evolve faster than bacteria. Given the high level of phage specificity towards the bacterial host and the relatively narrow host range exhibited by most phages, the impact on the physiological (health-protecting) microbiome of individual patients during PT is minimal [19,113].

Bacteriophage-based preparations are currently available in various forms, including monophages or polyphages [11]. Monophage therapy involves the use of a single type of phage as a therapeutic agent. The equivalent of monophages are single-receptor phages that recognize the single, specific receptors on the surface of bacterial cells. The narrow host range of monophage therapy or single-receptor systems limits their use. In this procedure, it is important to match the pathogen(s) and phage(s). Therefore, it is usually used to develop experimental models of PT. This technique can also provide a proof of concept when designing and testing phage preparations. A good alternative and a promising approach (against different infections) seems to be polyphage therapy based on phage cocktails, which can be used to eliminate many bacterial strains of a single species, various bacterial species, or a certain bacterial strain. In this method, different bacteriophages must be combined, which allows for a wide range of antibacterial activity and the extension of the host range. The processes for preparing and purifying such formulations are complex due to the need to evaluate each ingredient of the cocktail and eliminate low-yield phages [11,19]. The dual-receptor phages show similarities to polyphage therapy. This system can be based on a combination of two phages, each recognizing a different receptor (e.g., phages SP21 and SP22 binding the outer membrane protein, OmpC, and LPS of the enetrotoxigenic strain of *E. coli* O157:H7) or single phages that utilize two different bacterial receptors (e.g., phages T4 and T2, recognizing LPS and OmpC, or surface protein and bacterial polysaccharide, respectively) [118,119,120,121]. This type of phage preparation also prevents the development of phage-resistant bacterial pathogens. The phages may be administered sequentially or simultaneously. The sequential applications of phage cocktails can reduce the bacterial population density and the emergence of phage-resistant bacteria. However, simultaneous phage application is equal to or better than sequential administration, taking into account the reduction of the bacterial population without visible differences in terms of minimizing resistance (e.g., simultaneous application of a cocktail of SP21 and SP22 phages prolonged the emergence of phage-resistant *E. coli* bacteria to 30 h). Such a therapeutic effect results from the fact that even if bacterial resistance appears, the next phages from the cocktail will fulfill their treatment function. Therefore, the simultaneous system works especially well as short-term therapy, and the sequential strategies are more effective in treatments over longer time scales [56,59,118,122].

At the present time, different methods are being sought to increase the stability of phage products, their systemic therapeutic effect, and their controlled release in the treated organism. Various routes of phage administration are available, i.e., oral, topical, intravenous, intraoperative, intrarectal, and nebulization. However, the simplest and safest route, which is oral administration of phages, is not always suitable [26,46,67,123]. The problem with this form of phage application is their poor stability in gastric acid conditions and insufficient retention time in the intestine, which requires frequent administration of free phages [124]. In addition, divalent metal ions, which are necessary for bacteriophage replication, can be blocked by bile salts and intestinal carbohydrates. It was also found that oral phages cannot play a systemic therapeutic role due to their limited ability to get from the digestive system into the systemic circulation. Intravenous injection of phages partially solves this problem, but it also has its drawbacks (e.g., administration and supervision by healthcare professionals, potential for cross-contamination) [19,26]. Therefore, in some respects, microneedle-based PT (using the transdermal delivery system) is a better option. The microneedles can completely penetrate the skin and deliver phages. In addition, the above procedure is also an important parenteral (extra-gastrointestinal) delivery system with the possibility of self-administration of phages (by patients). In turn, the disadvantage of such phage application is the rapid elimination of phages and the need for frequent administration [19,125].

Another method that protects phages against unfavorable conditions in the stomach (i.e., low pH, presence of digestive enzymes such as pepsin, protease, and lipase) is their encapsulation (e.g., liposome-, polymer-, and electrospun fiber-encapsulated phages) [19,126]. Positively charged liposomes protect phages against stomach acids, extend their retention time in the intestines (acting as promoters of mucus adhesion), and increase tissue adhesion. The activity of the liposomes can also be modified to specify their function. Their disadvantage is the formation of larger conglomerates through mutual adhesion or fusion. This causes an increase in the size of liposomes, which limits their stability and results in non-specific phage interactions with target cells [127,128,129,130]. Among the modified biopolymers used for phage encapsulation, the most common is alginate. The acid stability of alginate is increased by the addition of, e.g., chitosan, pectin, neutral, or guar gum. Generally, chemically modified biopolymers are used for oral application of phages to treat gastrointestinal tract infections. Phages administered in this way do not activate pro-inflammatory cytokines and do not stimulate the production of antibodies. However, the polymers that entered the bloodstream stimulate phagocytes, which leads to their elimination [131,132,133]. Electrospun biopolymer fibers with a three-dimensional structure are also a very good carrier for phages, which can be wrapped on their surface or inside. Phages encapsulated in electrospun fibers can be used not only to capture and kill bacteria but also to detect, identify, and immobilize target microorganisms. The fibers enable the continuous transfer of bioactive ingredients. The release of phages through, e.g., fiber expansion or dissolution of the polymer is controlled by the selection of appropriate materials (e.g., mixed fiber polymers). The disadvantage of this system is the possibility of destruction of the phage as a result of rapid dehydration during electrospinning [134,135,136].

Phages are also well tolerated immunologically and can interact with the host’s immune system. Phages have adapted very well to this type of interactions, e.g., the *E. coli* T4 phage has immunoglobulin-like domains in capsid proteins that interact with mucins and surface-located glycoproteins on mammalian epithelial cells [137]. Similar domains belonging to the Ig superfamily can be found in many different phage families [138,139]. This results in an increased content of phages in the mucosal layers. In turn, their binding to mucous membranes increases the susceptibility of some bacteria to phage lysis. In this way, a strain-specific mucosal phage barrier is created, protecting the host against bacterial invasion [137,140]. However, the action of phages associated with mucous membranes is not limited to the epithelium. Phages use cell transcytosis and paracytosis in inflammatory sites to cross the human intestines and translocate into the circulatory system (e.g., T1 phage introduced directly into the small intestine penetrates the intestinal drainage of lymph and blood). The rate of such transit is 3.1 × 10^7^ particles per day. The circulating phages are active, which determines their participation in the defense of host cells against bacterial invasion [141,142,143,144].

### 4.2. Potential Disadvantages

PT has relatively few disadvantages. This is largely due to a much better understanding of phage biology and higher standards of medical research [19,98]. The main disadvantage relates to the identification of an appropriate lytic phage with a high virulence and a broad spectrum of bacterial hosts (i.e., species and strains) to tailor their suitability for different patients suffering from infections caused by various bacterial strains. Another concern during PT can be temperate phages, which are able to modify the bacterial genome by incorporating their genetic material (by lysogenic transformation) into the genome of infected cells (increasing the pathogenic potential of the host) and obtaining (by transduction) new genes from infected bacteria such as, e.g., virulence genes or antibiotic resistance genes (which are then transferred to new host cells) [66]. Therefore, the use of such phages during treatment should be avoided, especially before their sequencing and genetic modification, which can increase their safety during application.

Sometimes bacteria become resistant to phages, resulting in the appearance of phage-insensitive mutants [145,146]. Studies performed indicate that mutations in bacteria leading to resistance to a particular phage occur less frequently than to antibiotics. A phage-resistant bacterium can emerge at 10^7^ cell divisions. In contrast, one mutation toward antibiotic resistance occurs per 10^6^ cell divisions. However, the probability of a simultaneous mutation to a phage and an antibiotic is even lower—per 10^17^ cell divisions [147]. The resistance mechanisms can be very diverse and include, e.g., degradation of phage DNA (by the CRISPR-Cas system, restriction modification system, and other strategies), blocking of phage DNA transcription and replication or phage protein synthesis (using antiphage signaling systems and abortion infection systems), and modification of bacterial receptors resulting in the inhibition of the adoption of phages to the host cells [148,149]. However, this is not a major problem considering the large variety of phages in the environment, which allows us to find a suitable virus that will lyse the resistant bacteria. A very good example is the MDR *Acinetobacter baumanii* showing resistance to phage, administered intravenously to a treated patient, and the isolation of a new phage (from wastewater) with activity against the above bacteria [81]. It is also possible to apply phage cocktails (including several different virus strains) instead of monophage or phage-derived therapeutic proteins, which can limit phage resistance [19]. This underlines the possibility of using phages in the treatment of atypical and resistant infections, as well as personalized therapies.

In addition, a long-term PT can result in anti-phage antibody production that sometimes interferes with the treatment applied. A limiting effect of neutralizing antibodies has been observed in a bronchiectasis patient with refractory *Mycobacterium abscessus* lung disease. The patient was treated intravenously for 6 months with an active phage cocktail, which was safe for him and reduced the *M. abscessus* sputum load tenfold over a month. In turn, after two months, the patient developed a strong humoral response associated with the production of IgM and IgG neutralizing phage antibodies, which limited the effectiveness of the therapy [37]. On the other hand, another study analyzed the humoral immune response (i.e., the production of anti-phage IgM, IgG, and IgA antibodies) and the neutralizing properties of the antibodies to the applied (orally or topically) *Staphylococcal* MS-1 Phage Cocktail (against methicillin-resistant *Staphylococcus aureus)* in patients undergoing experimental PT. However, the presence of antiphage antibodies did not correlate with the unsatisfactory clinical results of this type of treatment. In addition, a negative therapeutic effect was observed in some patients who had relatively low serum anti-phage antibody production during the administration of the phage preparation, before therapy, and also during the whole treatment. For comparison, in the case of patients who showed the highest level of antiphage antibodies and the highest antiphage activity in their sera, the treatment was completed with very good clinical results or even full recovery [38]. Most studies to date indicate that phages can induce a humoral immune response in the human host. However, the interpretations about the strength of this response are often vague and confusing. Therefore, further research on phages and the immune response dependent on them is needed to increase the efficacy of PT (Table 2).

Despite the therapeutic benefits of phages, this type of treatment may also cause some social anxiety. This is due to the fact that not all patients are familiar with phages and the therapy based on them. It is therefore imperative to accept phages as good, non-pathogenic viruses, especially in Western countries [150].

## 5. Challenges and Future of Phage Therapy

Phage-based therapy as a living therapeutic agent is a promising tool for modern medicine. It provides an alternative approach to combating resistant pathogens. However, as a potential routine form of treatment worldwide rather than a medical experiment, it must be preceded by extended scientific and clinical research. And the misconception of phages as potential “causative agents” of human infections should be addressed and eliminated. The introduction of this type of treatment on a global scale may have measurable benefits for improving the health condition of millions of people who are infected with MDR bacteria each year [46,58,67].

Zoonotic pathogens that enter the human food chain are also a challenge for PT, representing a significant medical problem worldwide. The increasing demand for meat creates the need to develop this alternative (to antibiotics) form of prevention and treatment of bacterial infections, which are often resistant to several antibiotics. Unfortunately, in UE, there is no regulatory framework for the introduction of phages, e.g., into water or feed, as part of washing procedures for farm animals, or in modified packaging in slaughterhouses. Regulations limiting the release of phages into the environment must also be taken into account. Therefore, it is important to design phage interventions in the future with all safety standards preventing the release of phages outside livestock farms [13].

PT involves the identification and cultivation of a patient’s clinical isolate responsible for a given type of infection in order to test its susceptibility to phages. The knowledge is essential for physicians, who should have access to several phages targeting a specific strain of bacteria, preferably based on phage biobanks. Large phage collections are a prerequisite for high-throughput screening and typing of the appropriate phage or phage cocktail that matches a given bacterium. This is especially important in the case of personalized medicine. The treatment requires the presence of a large phage bank containing the produced phages, which, after selection, can be prepared in the form of a medicinal product intended for a specific patient [26,40,151].

Considering the constantly growing resistance of bacteria to antibiotics, there is a real need to continue the search for new phages and their adaptation as potential therapeutic agents. However, the commercialization of phage preparations by pharmaceutical companies is not very frequent due to the high financial costs that can be used in a different way than PT. In addition, there are also difficulties associated with patenting phage-based products. Lack of regulatory approval for such treatment on a large scale (by, e.g., the FDA) is also a very big problem closely related to the absence of flexible approval mechanisms [5,14,45,46]. Therefore, further steps are needed to overcome regulatory hurdles and achieve common phage-based therapeutic guidelines.

In the future, huge technological progress may solve the problem of patenting phage preparations. This can contribute to increasing the specificity and availability of PT. Phages can also be modified using genome editing techniques (e.g., sequencing, CRISPR/Cas-based phage engineering, homologous recombination, phage genomic DNA assembly) to obtain preparations capable of killing only antibiotic-resistant bacteria (without affecting the commensal microflora of the treated patient). Such unique phages or phage cocktails can be more easily patented and commercialized. The need for readily available phage products can be met by phage libraries established by various research teams and PT centers around the world. The production of safe phage drugs, whether on a small scale for personalized therapy or on a larger scale, requires validated quality control measures. In any case, the sterility, stability, and absence of endo- and exotoxins or other harmful impurities in the therapeutic preparation must be considered [152,153,154,155,156].

Whatever the fate of phages as living drugs and “cures for everything” in the future, most experienced researchers and experts believe that this form of treatment will never replace antibiotics. The phage-based therapeutic strategy can be used in combination with antibiotics or as a last line of defense for patients suffering from infections that have not been effectively treated with any other available therapeutic method. Given the rapid increase in the number of life-threatening MDR infections (resulting from the widespread use of antibiotics) in recent years, there is a need to revisit the potential role of PT and other alternative treatments versus antibiotics in combating antibiotic-resistant strains. The therapy based on phages certainly deserves a second chance in Western medicine [67,154].

## 6. Conclusions

Given the steadily growing incidence of MDROs, there is great interest in exploring non-antibiotic treatment options [3,6,48]. Nowadays, PT is the focus of interest in many countries (including, e.g., Poland, Belgium, Australia, or the USA). Huge collections of phages characterized molecularly, biophysically, and genetically are being developed, ready to be used in human and animal therapy to save lives [44,46]. Phages can definitely help in the fight against bacterial pathogens that are resistant to most, or virtually all, antibiotics.

In recent decades, PT has been modernized and re-emerged as a potential treatment for resistant infections, mainly for two reasons. Firstly, the resistance of bacterial pathogens to available antibiotics has increased significantly, and the number of new antibiotics has increased only slightly. Secondly, the number of clinical trials funded by various agencies and pharmaceutical companies, as well as biotechnological start-ups, has increased [22,37,81,92]. This provides the basis for the development of alternative or complementary anti-infectives to conventional antibiotics.

This therapy is currently very challenging in terms of aspects such as, e.g., the lifetime of single phages or combinations of phages, antagonism and synergy of phages and/or antibiotics, pharmacokinetics, pharmacodynamics, pharmacogenomics, as well as host responses to phage preparations. The first and most important step in this approach is to develop a large and widely available phage bank and effective mechanisms to screen them for target bacterial isolation (that are resistant to antibiotic treatment). Another is the elimination of bacterial infection (by cell lysis) in the sick patient, preferably without causing bothersome side effects or disturbing the natural microflora [19,46,123].

Phages are generally considered safe for humans, which is due to the fact that they cannot multiply in the cells of the treated organism and are excreted by the patient’s kidneys once the antibiotic-resistant pathogen is killed. However, phage lysates may contain many types of harmful products hazardous to health and life, mainly endotoxins (LPS) from Gram-negative bacteria and protein toxins produced by bacterial pathogenic strains. Hence, there is a constant need to improve methods of isolation, enrichment, and purification of phage preparations to highly effective and safe forms useful in human and animal therapies [57,97,156].

It can be expected that with technological advances (involving, e.g., genetic engineering, synthetic biology, throughput sequencing, and metagenomics), it will be possible to obtain phage products in specialized GMP (Good Manufacturing Practice) facilities. Although the isolation of natural phages from many pathogens is very simple, there are still many species of bacteria for which phages are rare or not available at all [67]. This means that it is necessary to identify new sources for obtaining and isolating phages with a wide host range.

In the future, PT may represent a new and widespread approach to combating resistant pathogens [22,58]. Therefore, logistical and regulatory obstacles must be overcome for PT to become an alternative and generally available form of treatment for patients who have failed antibiotic therapy. Additionally, further clinical studies are still needed to complement clinical data on phage biology, dosing of phage preparations, and PT administration methods.

## Figures and Tables

**Table 1:** General overview of phage testing, discovery, and phage preparations [19,22,57,58,59,60,66,67,69,94,95].

Basic Steps in Phage Research	Characteristics/Description
Phage isolation	Types of environments (oceans, lakes, rivers, soil, plants and animals, sewage treatment plants, waste from animal or human communities depending on the bacterial pathogen), phage collections/banks, where and when the isolation took place, and publications documenting the research on phages and their original isolation. The presence of phages is characterized by cloudy to clear suspensions containing specific hosts or the formation of clear or cloudy phage spots on agar plates with the growth of host cells.
Phage enrichment	Repeating multiple rounds of adsorption-elution-amplification procedures with competing receptors to obtain high titers of phage capable of effectively infecting bacteria.
Phage purification	Centrifugation, repeated filtration, and chromatography (including ultrafiltration, dialysis filtration, ion exchange chromatography, affinity chromatography, and size exclusion) upon completion of phage propagation by the host bacteria in the production medium. The methodology strictly depends on the type of phage. Contamination of the preparation with endotoxins and proteases should be avoided.
Phage identification and characterization	Virion characterization: morphological, physical, and chemical. Growth parameters: host range, adsorption rate, latency, burst size, and thermal, pressure, UV, chemicals, or pH stability. Visualization: transmission- and scanning electron microscopy (TEM, SEM), Cryo-EM, and atomic force microscopy (AFM).
Genomic analysis	Gene functional annotation, prophage, regulatory elements, host predictions.

**Table 2:** Positive and negative aspects of phage therapy [19,22,26,37,49,56,59,64,66,67,70,81,96,98,102,112,130,133].

Phage Therapy	
**Major Advantages**	**Description/Comments**
Safe and effective bactericidal agents	Bacteria successfully infected with lytic phages are killed, which prevents the evolution of bacterial resistance.
Low inherent toxicity	Inherently non-toxic phages despite the presence of proteins and nucleic acids.
Auto “dosing”	Establishing the phage dose by the phages themselves depending on the density of the bacterial host.
Minimal disruption of commensal microflora	The high level of phage host specificity limiting damage to other bacterial species and genera.
No cross-resistance to antibiotics	Efficient clearance and treatment of bacterial infections resistant to antibiotics.
Various formulations and administration of phages	Monophages, cocktails, microneedle-based phages, liposomes, polymers, and electrospun fibers—encapsulated phages.
Immune tolerance	Interactions with the immune system of mammals result in the formation of a strain-specific mucosal phage barrier as a host’s defense against bacterial invasion.
Low costs	Relatively low production costs with technology improvement.
Phage-Antibiotic Synergy (PAS)	Synergistic phage-antibiotic combinations stimulating virulent phage growth.
Alternative treatment to antibiotics	The treatment of patients with renal impairment, allergy to antibiotics, or immunodeficiency.
Personalized therapy	“Magistral phage medicines” produced at a pharmacy according to a physician’s prescription for individual patients.
Biofilm clearance	Lysis of bacterial layers within the biofilm structure and depolymerization of capsular and structural polysaccharides by depolymerases.
**Potential disadvantages**	**Alternative solutions**
Phage selection and safety	Requirements: “obligately lytic” phages (not temperate), unable to display lysogeny, released from infected cells via lysis, stable under typical storage conditions and temperatures, fully sequenced to confirm the absence of unwanted genes, e.g., toxins, to avoid modification of the bacterial genome, changes in their phenotype, and increased toxicity.
Phage host-range limitations	Narrow range of bacterial hosts (several strains/species) limiting treatment procedures. The possibility of using phage cocktails or a combination of phages and other antibacterial agents broadens the lytic spectrum of phage products in relation to the spectrum of individual phage types.
Phage resistance mutants	A frequent phenomenon that can result in therapy failure, which can be circumvented by the treatment methods indicated above and searching for new phages from the environment.
Phages as a not-unique pharmaceutical	Live biological agents based on proteins capable of evolving during production and use. For some phage therapy protocols, the use of highly purified phage preparations is required.
Anti-phage antibodies interfering with treatment	Possible influence of anti-phage antibodies on the results of therapy, depending on, e.g., the route of administration, dosing schedule and duration of treatment, and immunogenicity of phages.
Cultural unfamiliarity with phages	Misconception of phages as potential viral pathogens causing human diseases. Currently, several phage products have been classified as GRAS (Generally Regarded As Safe) by the FDA.

## Data Availability

Not applicable.

## Abbreviations

MDR: multi-drug-resistant; MDROs, MDR organisms; AMR, antimicrobial resistance; FDA, Food and Drug Administration; CDC, Centers for Disease Control and Prevention; WHO, World Health Organization; PT, phage therapy; PK, pharmacokinetics; PD, pharmacodynamics; MOI, multiplicity of infection; PPT/APT, passive or active type of PT; IR, immune response; PTC, PT center; IND, Investigational New Drug; EMA, European Medicines Agency; EIBMV, Eliava Institute of Bacteriophage, Microbiology, and Virology; HIIET, Ludwik Hirschweld Institute of Immunology and Experimental Therapy; EU, European Union/endotoxic unit; FAMHP, Federal Agency for Medicines and Health Products; IPATH, Innovative Phage Applications and Therapeutics; TGA, Therapeutic Goods Administration; ATCC, American Type Culture Collection; PHE, Public Health England; NCTC, National Collection of Type Cultures; CR, Creative Biolabs; LPS, lipopolysaccharides; PFU, plaque-forming units; CFF, cross-flow filtration; OmpC, outer membrane protein; TEM, transmission electron microscopy; SEM, scanning electron microscopy; AFM, atomic force microscope; CRISPR, clustered regularly interspaced short palindromic repeats; GMP, Good Manufacturing Practice.
